# Vagrants

**Published:** 1900-09-22

**Authors:** 


### The Hospital

A Journal of

The Medical Sciences and Hospital Administration.

Vol. XXVIII.—No. 730.] Edited by Sir Henry Burdett, K.C.B., and Solomon C. Smith, M.D., M.R.C.P. [September 22, 1900.

### VAGRANTS.

It is a matter for regret that the abundance of papers annually presented to the British Association for the Advancement of Science, and the amount of space required even for condensed reports of them, render it practically impossible for newspapers to furnish more than brief abridgments of a large proportion; or to furnish at all, save in very exceptional instances, the discussions to which they have given rise, and which, as they would presumably contain the opinions of persons who had regarded the questions at issue with some attention, and from different points of view, might not infrequently be of greater aggregate value than even the communications which had called them forth. There could scarcely be a better illustration of this than is afforded by a paper on "The Treatment of the Tramp and the Loafer," read to the Association on the 11th instant by Mr. W. H. Dawson, and containing proposals for the practical suppression of the nuisances to which it refers; nuisances which, as every medical officer of health is perfectly well aware, are prejudicial, not only to the moral and industrial welfare of the community, but also, as being frequent channels for the diffusion of epidemic and other forms of contagious disease, to its sanitary welfare as well. A belief is commonly entertained in this country that the origin of vagrancy may be traced to the dissolution of the feudal system and the suppression of the monasteries, by which a great part of the rural population were deprived of their means of support; but this belief, even if it be true in some degree, is at any rate only a very partial expression of the facts. Vagrancy arises from, or depends upon, a particular type of character; and, although varying social or legislative conditions may have sufficed to modify this type at different periods, they have neither created it, nor will they, except by very slow degrees, suffice for its extinction. It is not generally known that Martin Luther was among the first systematic writers on vagrancy, or that the "Liber Vagatorum," or "Book of Vagabonds and Beggars," which he edited in 1529, and of which an English translation was published in 1860, contains a description, which Mr. Dawson himself could not surpass in minuteness or fidelity, of most of the forms of vagrancy which are familiar to us in the present day. Mr. Dawson described the class as an "ever growing" one, but we have no evidence on the question whether its growth increases in greater proportion than population. The census of 1891 showed the number of vagrants to be about 60,000, and the time for another determination of this number is drawing near. The class, we ought not to forget, is mainly constituted by, or recruited from, persons whose instincts and peculiarities, at one period of the history of the human race, would have been counted to them for righteousness. These instincts and peculiarities, controlled by law and disciplined by education, lead men to explore the earth and to possess themselves of its treasures; and it is only when suffered to run to seed, if we may so phrase it, that those under their guidance become chiefly conspicuous as idlers or criminals. John Cary, a Bristol merchant, through whose wise and philanthropic efforts the first "workhouse" for the poor was established in his native town in 1697, says of tramps and beggars "that he who observes the fatigues used by them to make themselves seem objects of charity, must conclude that they take more pains than an honest man doth at his trade." But it is not only, nor even chiefly, in relation to the misdirection of effort that their numbers are to be deplored. They afford a perennially flourishing bad example to young people of analogous tendencies with whom they may come into contact; while their habitual aggregation in casual wards or lodging-houses, and the effect of their talk and action upon the comparatively small number of *bonâ fide* "travellers" in real search of work, constitute a danger as well as an annoyance to the public.

Mr. Dawson advises that the casual ward and the common lodging-house should alike be closed, and that in England, as in Germany and Switzerland, idle persons without visible means of support should be committed to real "workhouses," and compelled to labour in earnest under strict discipline for such a period as might afford them an opportunity of reformation, or permanently if this opportunity were neglected. The case is one in which, when its conditions are fully known, indulgence will appear to be misplaced. The possible effect of the migration of tramps from localities affected with epidemics, such, for example, as Glasgow, is enough to show even to the least thoughtful how powerfully the class may contribute to the extension of formidable disease, while it is certainly not too much to say of them, on the other hand, that their existence and their freedom are not attended by any compensating advantages. They render no services, and they produce nothing, except offspring who, in due course, will come to inherit the vices and to tread in the footsteps of their parents.

